# Exploring the feasibility of eHealth solutions to decrease delays in maternal healthcare in remote communities of Ghana

**DOI:** 10.1186/s12911-017-0552-z

**Published:** 2017-12-02

**Authors:** Pedro Pagalday-Olivares, Bengt Arne Sjöqvist, Jessy Adjordor-van de Beek, Samuel Abudey, Ants R. Silberberg, Ruben Buendia

**Affiliations:** 1 Department of Signals and System, Chalmers University of Technology, -412 96 Göteborg, SE Sweden; 2 UNiTED Projects, P.O. Box 216, Kpando, Volta Region Ghana; 3 District Health Directorate, Kpando, Volta Region Ghana

**Keywords:** eHealth, mHealth, Maternal health, Maternal mortality ratio, Skilled maternal care, Rural communities, Ghana

## Abstract

**Background:**

Despite the introduction of the Millennium Development Goal to reduce maternal deaths from 400 to 100 per 100,000 live births, the proportion of maternal deaths is still much higher in most developing countries like Ghana. Various interventions have been implemented in Ghana that focus on increasing skilled maternal care. These are especially needed in rural areas. EHealth has the potential to contribute to reducing the challenges in maternal healthcare (MHC) that poor areas suffer. This paper analyzes the potential of eHealth solutions to improve maternal health in rural Ghana as well as the challenges to their implementation. The work was carried out in cooperation with the local health directorate of Kpando Municipality, one of the administrative areas in the Volta Region.

**Methods:**

The study is focused on remote peninsulas and islands in Kpando Municipality. Data was gathered through triangulated research methods. Maternal health challenges were identified using the Three Delays Model for MHC. The three delays are delay in seeking care, delay accessing health facilities, and delay receiving adequate care. Challenges to the implementation of eHealth solutions in remote communities were analyzed using the Drury’s 5C eHealth model for developing countries. The 5Cs correspond to *context*, *community*, *capacity*, *connectivity*, and *content*.

**Results:**

The results show that financial dependence of women, a decision-making process based on previous experiences and traditional beliefs, competitiveness between facilities, organizational loopholes, lack of equipment, and geographical situations directly influence MHC outcomes. EHealth solutions, thanks to the high number of health workers with basic IT skills, have high potential to reduce MHC delays. However, poverty, cultural beliefs, organizational issues, connectivity, and lack of human resources were identified as main challenges to the implementation of eHealth solutions.

**Conclusion:**

In Ghana’s rural areas the three delays proposed in the model affect the outcomes of MHC. These delays are influenced by socio-economic status, access to facilities, and quality of care. EHealth solutions show great potential to reduce the delays. Based on the 5C model, a mHealth solution aiming to improve guidance during pregnancy was outlined.

## Background

A maternal death is defined by the World Health Organization (WHO) as: “*death of a woman while pregnant or within 42 days of termination of pregnancy, irrespective of the duration and site of the pregnancy, from any cause related to or aggravated by the pregnancy or its management but not from accidental or incidental causes*” ([1], pp.-156). Each day in 2015, 830 maternal deaths occurred worldwide from largely preventable causes like eclampsia, obstructed labor, and unsafe abortions [2].

In the year 2000 world leaders adopted the United Nations Millennium Declaration. Eight time-bound targets were set with deadlines in 2015. Millennium Development Goals (MDGs) aimed to reduce extreme poverty and promote peace, human rights, and security. MDG 5 focused on reducing the maternal mortality ratio (MMR) from 400 to 100 maternal deaths per 100,000 live births in the period between 1990 and 2015 [3]. As MDG 5 has not been met in many countries, continued action is needed [4]. To build on the MDGs and achieve what they did not, 17 Sustainable Development Goals (SDGs) and 169 targets were announced in the draft 2030 agenda. Maternal health falls under goal 3, targets 3.1 and 3.7. The goal is “*to reduce MMR to less than 70 per 100,000 live births*” and “*ensure universal access to sexual and reproductive healthcare services, including family planning, information and education and the integration of reproductive health into national strategies and programs*” ([5], pp.-16).

Ghana is one of the countries that has adopted the draft 2030 agenda. This West African country has a population of 27 million (estimated in 2015). Ghana is divided into ten administrative regions, 138 districts, and 58 councils [6]. The present study focuses on remote peninsulas and islands of Lake Volta which search for healthcare in Kpando Municipality, located in the Volta Region. Most peninsulas considered belong to Kpando municipality, otherwise, the islands do not belong to any municipality. The Volta region extends east of Lake Volta, the world’s largest man-made reservoir by surface area and the fourth largest by water volume. Almost 30% of the land of Kpando Municipality is submerged by the lake, resulting in many remote communities—peninsulas—on the lakeside [7]. A census conducted on seven of the islands situated close to Kpando confirmed 1092 inhabitants [8]. Services provided to these communities, like maternal healthcare (MHC), are limited.

Healthcare in Ghana is decentralized. The Ghana Health Service (GHS) is responsible for the implementation of policies. Strategies and interventions in maternal health are based on the Safe Motherhood Initiative (SMI), a worldwide effort that was launched in 1987. This initiative consists of five pillars: antenatal care (ANC), delivery care, postnatal care (PNC), emergency obstetric care (EmOC), and family planning. The aim of the initiatives is to increase the number of deliveries supervised by skilled birth attendants (SBA) and increase the number of ANC and PNC attendants [9].

**Fig. 1:**
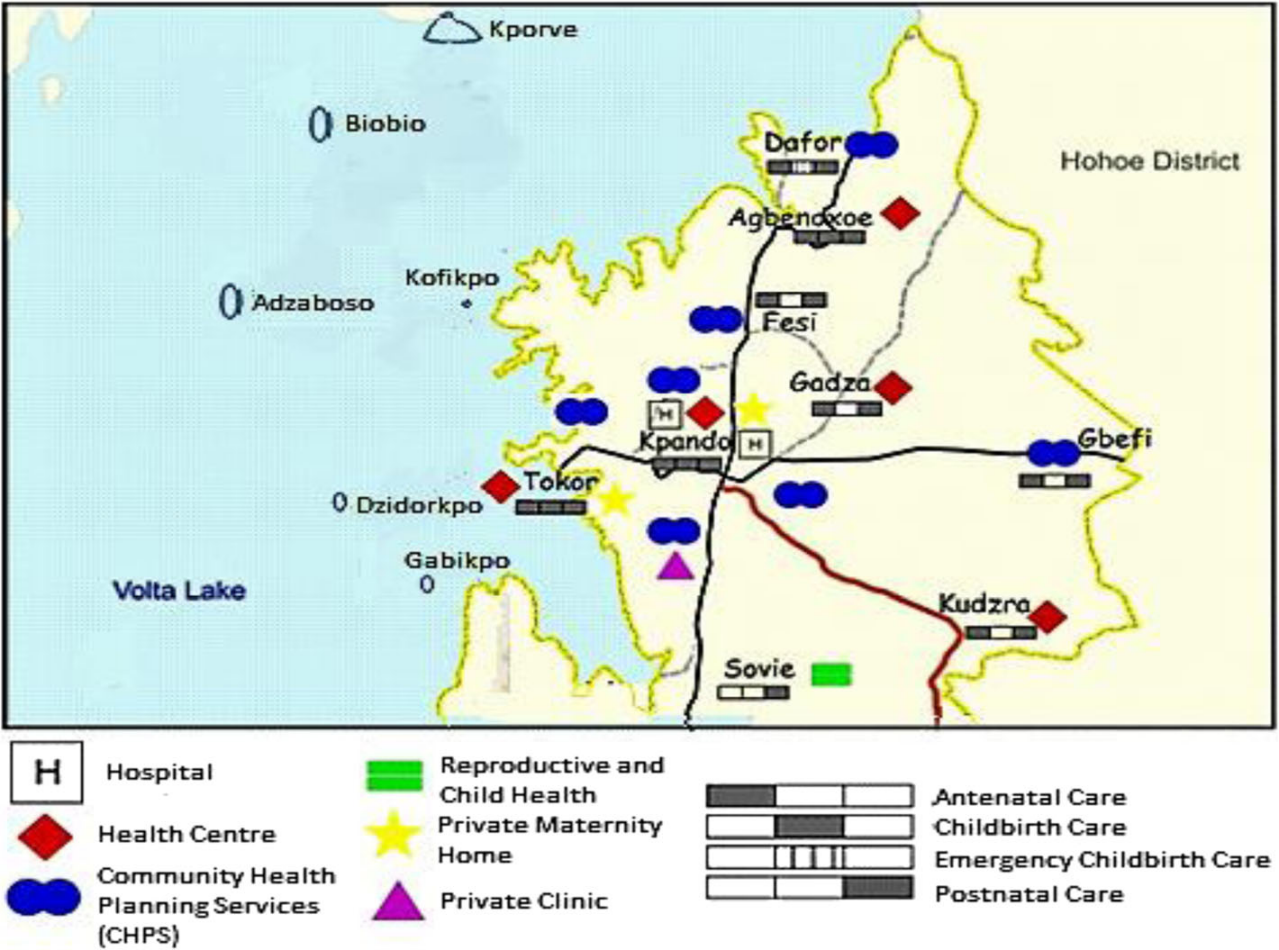
Map of maternal health care in Kpando

A map of the focus area of this study is shown in Fig. 1 Map of maternal health care in Kpando, which displays the available MHC facilities and service provisions in Kpando Municipality. Childbirth care is provided at five facilities: a health center, two hospitals, and two maternity homes. As the map shows, these facilities are located in urban Kpando and the remote areas of Torkor and Agbenoxoe. ANC is provided in all facilities except for the Sovie Reproductive and Child Health (RCH) facility. PNC is provided in all facilities. No MHC facilities are available on any of the islands included in this study [8].

The primary providers’ for the included remote communities are the Dafor Community Health Planning and Services (CHPS) zone and Agbenoxoe Health Center. At Agbenoxoe, community health nurses (CHNs) provide outreach ANC and child welfare clinics at irregular intervals in addition to the MHC services. The Torkor HC, CHPS zone, and Wisdom’s Maternity Home are the first points of care for women living on the islands included in this research. The HC and CHPS zones provide ANC and PNC, while all MHC is provided at the maternity home. In both areas considered, home deliveries assisted by traditional birth attendants (TBAs) are common [8]. TBAs are often untrained community members who do not have access to the appropriate knowledge and equipment. In both included areas, complicated deliveries are referred to one of the hospitals in Kpando [10].

### Skilled care attendance and MMR

The MMR in Ghana was reduced from 590 in 1995 to an estimated 358 in 2015. Hence, the MDG target to reduce MMR to 185 in 2015 has not been achieved [9]. Moreover, the MMR of non-institutional deaths remains unknown. It is estimated that more than 80% of maternal deaths occur in the communities or within 24 h of admission to a health facility [10]. Moreover, 80% of maternal deaths can be attributed to five treatable and preventable complications: hemorrhage, sepsis, eclampsia, obstructed labor, and complications of abortion [11]. The MMR in Kpando was 623 in 2010, reduced to 161 in 2013. However, it increased to 205 in 2014. The main causes were: cardiac failure, hemorrhagic shock, respiratory failure, and acute chest syndrome. Causes of mortality in Kpando and Ghana are different but might be related, e.g. cardiac failure can be caused by eclampsia [12], and obstructed labor and complications of abortion can lead to hemorrhagic shock [13]. It is difficult to compare MMR in Kpando with the rest of Ghana because of the lack of patient data available in Kpando: unlike health data elsewhere, the underlying conditions leading to maternal deaths in Kpando are unknown because only the last symptoms were reported [12, 13].

Deliveries supervised by SBAs are increasing in Ghana but there is a big disparity between urban and rural areas. For instance, the Greater Accra Region around the capital of Ghana recorded 92% of deliveries supervised by a SBA in 2014, in contrast, the Volta Region recorded one of the lowest of the country with less than 50% [9, 11]. On the other hand, the contraceptive prevalent rate (CPR) defined as “*the proportion of women of reproductive age who are using (or whose partner is using) a contraceptive method at a given point in time*” is the highest in the country, with 32% in the Volta Region ([9], pp.-44). In Kpando, the proportion of births attended by SBAs was increasing steadily from 70% in 2010, reaching a peak value of 87% in 2013. However, in 2014 there has been a decrease of 13% and an increase in deliveries attended by TBAs. The health directorate attributes this to the lack of midwives in most facilities [7].

The percent of women attending ANC for at least one visit was 90.9% across the country; however, in the Volta Region this number was below average, with 81.9% in 2013. In fact, only 67.7% of registrants attended all four visits (as recommended by WHO), but ANC attendance has actually increased in the region since 2013 [14]. In Kpando, most women, register for ANC services during the 2nd or 3rd trimester of pregnancy, and only 30% of registrants attended the all four visits.

### EHealth in MHC

The biggest challenges in MHC are shortage of healthcare professionals, equipment, transportation, and health services at the municipal level. Other challenges are a lack of structure, a lack of regulation and inadequate data to assess maternal mortality issues [9]. Nevertheless, Ghana has high quality of care where this is available, a national health insurance system, and well-recognized facilities for medical education and accreditation [14].

On the other hand, the government struggles to extend these services to rural areas, even with the introduction of CHPS zones two decades ago. The program coverage is affected by logistical problems, supervisory lapses, and resource shortages. The Ministry of Health is trying to bridge the inequity gaps between rural and urban areas and rich and poor, looking for new ways to improve the outcome of investments made in the health sector. New policies such as free MHC and incentives for health workers to work in remote areas have been implemented [15, 16].

In 2003, as part of an attempt to optimize resources, the country started to consider eHealth as a way of improving healthcare [17]. Through this work, the term eHealth is used as defined by WHO in the WHA58.28 Resolution, i.e. *“eHealth is the cost-effective use of information and communication technologies (ICT) in support of health and health-related fields, including health-care services, health surveillance, health literature, and health education, knowledge and research”* ([18], pp.-1). EHealth has the potential to enhance healthcare systems’ capacity in developing countries [19]. Ehealth solutions are new healthcare delivery models capable of making qualified healthcare accessible to underserved areas—remote areas as well as areas lacking on-site medical expertise [19, 20]. In addition, eHealth solutions can also reduce costs for health service delivery, maintenance, and support. This makes them a viable and affordable way to improve healthcare in rural Ghana [21, 22].

In 2010 The Ghana National eHealth Strategy was published with the goal to: “*Harness the potential of Information and Communication Technology to improve the health status of people living in Ghana*” ([23], pp.-35). The report identified eHealth as a potential solution to several problems: large inequalities of access to healthcare among regions, weak referral, poor emergency systems, the increase of non-communicable diseases, and the lack of medical personnel [23].

Regarding the implementation of eHealth solutions, the identified challenges were: lack of ICT infrastructure in most facilities, low IT literacy, and lack of funding for ICT infrastructure. Local stakeholders cannot afford the increased cost of eHealth solutions [23].

In recent years, two eHealth solutions to improve maternal health have been piloted in different areas in Ghana. The first one aimed to send reminders by SMS to pregnant women [24]. The second one was a smartphone app to improve the knowledge of CHNs working in remote areas [25]. However, according to local authorities, no previous research has been done on implementing eHealth solutions in the study areas. Ehealth as it relates to our study encompasses telemedicine and mHealth, including a computerized decision-support system.

### Objective

This paper assesses the feasibility, in terms of potential of and requirements, of eHealth solutions to improve maternal healthcare in remote areas of Kpando, Ghana. Improvement in maternal care is approached as a reduction of the delay in receiving adequate maternal healthcare.

The geographical scope of this article is the remote peninsulas and islands in Lake Volta whose inhabitants seek healthcare in Kpando Municipality. Factors that influence skilled maternal care attendance and eHealth implementation were identified through a variety of data collection methods.

## Methods

The current MHC situation in Kpando, Ghana, with a specific focus on remote communities and the potential for eHealth solutions to decrease delays in maternal healthcare, was analyzed by triangulating a variety of research methods and sources. The research methods were: literature review, semi structured interviews, focus group discussions, and ICT infrastructure assessment.

The selection criteria for study areas were communities without easy access to qualified MHC whose inhabitants seek healthcare in Kpando Municipality. This resulted in the six islands and five peninsulas of Lake Volta plus an additional remote community (Agbenoxoe). Agbenoxoe has a unique type of care, with weekly community visits and intensive outreach programs to remote areas. Facilities that provide different types of MHC from our study areas and were willing to collaborate were selected through purposive and convenience sampling. Following these criteria, the first points of care available for islands and peninsulas were included. Additionally, facilities that provide outreach MHC and collaborate with TBAs in the communities were selected.

### Use cases

In order to get a deeper insight into the role of each stakeholder in MHC, use cases of typical pregnancies in the area were developed using the results of semi structured interviews, literature review and focus groups where pregnant women and health care workers explained typical cases of pregnancies in the area. Each use case includes the role of each of the stakeholders involved. The causes that could lead to maternal deaths were organized according to the TDM model. This resulted in the list of challenges for MHC in the area. The use cases also led to the identification of work routines and cultural beliefs that are challenges for eHealth implementation. In the use cases, details of events that occurred during pregnancy and childbirth for each stakeholder involved were mapped. Use cases are a suitable approach because they help to: exemplify typical pregnancies in the area, identify the weaknesses and strengths of the healthcare system, identify the reasons why the delays occur, and demonstrate how technology could help overcome the complications.

### Literature review

Three main sources of information were identified. Peer-review literature was the prime source for identifying impact assessment studies, reviews, and clinical outcomes of eHealth solutions in developing countries [24, 26–40]. Local, national, and international reports by NGOs, governments, and international agencies on maternal health provided quantitative information about skilled care attendance and maternal mortality. The third source of information consisted of reports of research carried out in the study areas; these reports are summarized in Table 1. The summary includes the stakeholders participating in each study.

**Table 1:** Stakeholders included in previous research in the study area

Stakeholders interviews	MHC in Kpando [35] (2013)	Influence of HIV testing on ANC attendance [32] (2013)	ANC utilization [36] (2014)	Delay in EmOC [37] (2014)	TBA influence in decision making processes [33] (2014)	Maternal health on the islands of Kpando [34] (2015)	Stimulating skilled care attendance in Kpando [31] (2015)
Pregnant women / mothers	20	18	14	21	22	60	11
Family members	–	–	3 husbands	–	–	–	4 (husbands), 5 (parents), 1 grandmother, 1 cousin
TBAs	13	–	–	7	6	6	2
Maternal health workers	26	5	10	6	3	3	12
GHS Directorate	1	–	1	1	–	1	1
NHIS Office	–	–	1	–	–	–	–
Additional Focus Group Discussion	–	–	–	1 with TBAs, 1 with mothers	1 with TBAs, 1 with mothers	1 with various stakeholders	1 with mothers, 1 with husbands, 1 with various stakeholders

Searches were conducted using Google and Google Scholar search engines, BMC Medical Informatics and Decision Making Process Journal, and ScienceDirect. Official documents from the UN and WHO were gathered from their online repositories. The keywords for the search were: maternal eHealth, eHealth in developing countries, maternal healthcare in developing countries, and impact assessment of eHealth solutions. Local reports and research related to maternal health were provided by the Health Directorate of Kpando and a local NGO at the study site.

Documents that contained impact assessment studies of eHealth in developing countries or presented eHealth solutions to improve MHC in developing countries were included. In addition, reports and recommendations on MHC, case studies of eHealth interventions, and detailed qualitative research on MHC in the study areas were also included. Each abstract was reviewed to determine if the document met the criteria.

### Semi-structured interviews

Semi-structured interviews [41] were conducted with healthcare providers at various levels at six facilities in Kpando Municipality to identify the MHC needs, as well as identify work routines in ANC, delivery care, and PNC. Fifteen health workers were each interviewed for about 20 to 30 min. Through these interviews, their perspectives on barriers and facilitators for pregnant women accessing MHC were gathered and work routines were identified. Finally, these health workers discussed the possibility of using eHealth solutions in their jobs and their willingness to do so.

### Focus group discussion

A focus group discussion [42] was organized with twelve MHC stakeholders: one representative of Ghana Education Service, one of National Health Insurance, two TBAs, three mothers from the islands, one representative of a private health facility that provide services to the islands, two midwives from GHS, one father from the islands and the Public Health Nurse from Kpando Municipality. The objective was to elaborate on the main pregnancy-related challenges and explore different solutions to the problem of poor access to MHC for the women living on the islands around Kpando. The challenges and their respective solutions were discussed and prioritized.

### ICT infrastructure assessment

In order to quantitatively analyze the telecommunication infrastructure, naturalistic observations [42] of the equipment in the healthcare facilities were performed. The focus was on mobile phone penetration among patients and ICT infrastructure in the facilities. Moreover, a network coverage test for the two biggest telecommunication operators in the area was performed, using the android application OpenSignal. Four different measurements were taken at each of 20 geographical locations at three-minute intervals for each operator. The average of the four measurements was used as an evaluation factor.

OpenSignal was used for quantitative analysis of the percentage of time the study areas have network access. OpenSignal automatically measure the time each location has access to network and provides a summary of it. The percentage provided in the results is the summary of 20 geographical locations, in each location the app was on for an average of 20 min, then turned off while moving to a different location. The total measuring time was 400 min.

### Theoretical frameworks for analysis

**Fig. 2:**
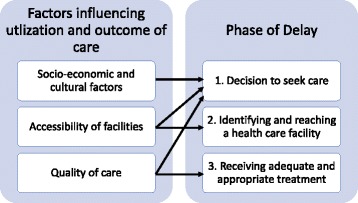
TDM Model

Two theoretical frameworks were used, the TDM and the 5C. The TDM, by Thaddeus and Maine [43], is a leading framework for assessing delays in maternal healthcare. Prompt, adequate treatment after the onset of obstetric complications will most likely result in a satisfactory outcome; however, a delay might lead to maternal death. Delays are not caused by one single factor, but by various interrelated factors. These factors are categorized into three groups (see Fig*.* 2).

**Fig. 3:**
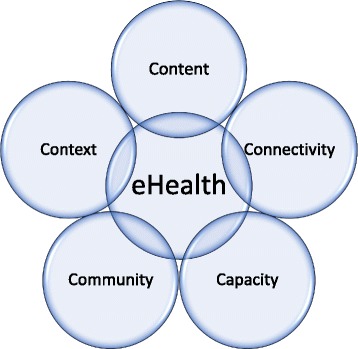
5C Model

The 5C conceptual framework by Drury [44], shown in Fig. 3, was used to structure research activities, outcomes, and recommendations. While the application of the TDM identifies factors that delay maternal healthcare, this framework analyzes the potential — and requirements — of eHealth solutions. The 5C framework was used to select the delay on which an eHealth solution would have the biggest impact. The five Cs are context, content, connectivity, capacity, and community. Information on the context, connectivity, capacity, and community was collected and analyzed during this research. The fifth C, content, will structure implementation research on the proposed solution. Challenges were placed on each category by researcher consensus.

All the gathered data led to the outline of a future eHealth solution to improve MHC. This strategic proposal should significantly decrease delays in maternal healthcare, improving the health status of mothers living in remote areas by addressing the main challenges of the healthcare system and the difficulties faced by mothers seeking access to healthcare. The prioritization is based on the relative importance that each participant of the study gave to the main challenges.

## Results

### Factors that influence delay

**Fig. 4:**
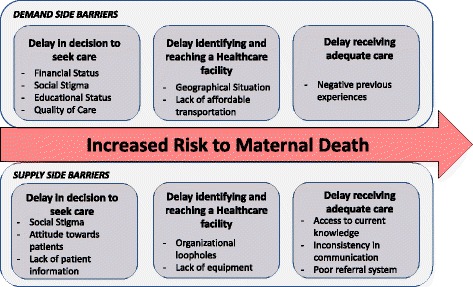
Identified Delays that increase risk of maternal death

Factors influencing delay in maternal healthcare according to the TDM model are summarized in Fig. 4. They are divided into three categories: socio-economic/cultural factors, accessibility of care, and quality of care. In addition, all factors influencing care are separated into those on the demand side (the patient) and those on the supply side (the healthcare provider). Following paragraphs further explain challenges identified in: Demand side through literature research, demand side through field research, supply side through literature research, and supply side through field research (i.e. semi-structured interviews and focus group discussion), respectively.

In previous research in the area, financial dependence of women, lack of education, and traditional beliefs were stated as the main of causes of delay in seeking care (first delay) on the demand side [45, 46]. Another key factor influencing the first delay identified in the literature was social stigma [10]. In [8, 47] pregnant women declared that distance to facilities, geographical situation and their economic status makes access to emergency care impossible at nighttime, hence, affecting the time it takes to identify and reach a healthcare facility (second delay). Finally, negative previous experiences came up as an influencing factor for the first and third delays [48]. As a result of these factors, among other problems, only 20% of women in the area go for ANC during the first semester. ANC visits are important to early detection and prevention of pregnancy related complications.

Some of these results were confirmed during the field research. On one hand, during the focus group discussion and in the semi-structured interviews with nurses, different stakeholders explicitly explained how lack of education led to maternal deaths. Furthermore, CHNs and stakeholders, confirmed poor road infrastructure, geographical situation, and the communities’ economic status as factors affecting the second delay during interviews and focus group. The lack of affordable transportation was a new factor that CHNs and mothers in the focus group identified during the field research. On the other hand, social stigma and negative previous experiences as factor influencing the quality of care (third delay) and first delay did not come up frequently during the interviews, but some stakeholders freely talked about it during the focus group.

On the supply side, the literature identifies social stigma, staff attitude towards patients, and competitiveness between facilities as factors influencing the first delay. Lack of equipment and human resourcers, organizational loopholes, and poor road infrastructure were identified as factors affecting the second delay, particularly in emergency cases [47, 48]. Finally, the literature reported that TBAs are a preferred choice of care due to their social recognition [10]—however, their lack of training affects the third delay.

On the field research, lack of equipment and human resources, organizational loopholes, poor road infrastructure and TBAs as prefered choice of care were confirmed during interviews and focus group discussions. A factor that was found only in the interviews was a lack of knowledge of patients previous complications, and how it affects the first delay. Finally, factors that were identified only in the interviews and affect the first and third delays were: inconsistency in communication, a poor referral system, and lack of access to current knowledge.

### Ehealth solutions to reduce delay

**Fig. 5:**
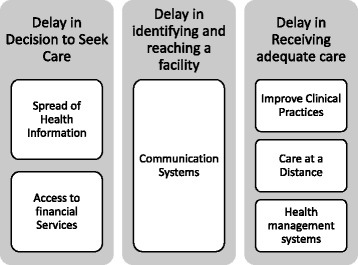
Potential of eHealth Solutions to reduce MHC according to TDM model

Figure 5 summarizes how eHealth solutions can help reduce delays in receiving MHC. The solutions are further developed in the following subsections.

#### Delay 1: Decision to seek care

As shown in Fig. 5, the literature reported two types of eHealth solutions with the potential to reduce the first delay. The first type is spreading health information using mobile phones, mainly through SMS or voice messages [24, 32]. Other alternatives use hotlines and call centers [28]. The aim of these solutions is to bridge the gap between communities, health facilities, and information services. Due to low mobile phone penetration in rural areas [49], some solutions rely on the use of the mobile phones of community volunteers. Previous studies showed the potential impact of these solutions to inform decisions to seek care [22, 50, 51]. In addition [32], showed that this intervention increased skilled birth attendance, but it did not find evidence of increased knowledge among recipients.

The second type of solution aimed at providing mobile financial services to pregnant women. It allows them to save and access financial services for MHC. Available studies outline the potential of fusing financial mobile services with healthcare [31]. However, the impact of these solutions has not been evaluated yet.

#### Delay 2: Identifying and reaching a facility

There is no general agreement about the best way to apply eHealth to reduce the second delay. Typical initiatives connect health workers in isolated areas with emergency systems through phone calls and SMS [34, 51]. Communication systems are effective at reducing maternal deaths and increasing skilled birth attendance. However, in order for these to be effective, availability of transport and good road infrastructure are also required [29].

#### Delay 3: Receiving adequate care

Three types of solutions for reducing the third delay have been identified: solutions that improve clinical practices, those that offer care at a distance, and health management systems. Electronic medical records (EMRs) and decision support systems are typical ways to facilitate improved clinical practice. EMRs systems have the potential to reduce medical errors as well as improving referrals and coordination between facilities. These systems are scaling up in developing countries. Decision support systems, including checklists and questionnaires, are effective at improving clinical and patient outcomes [33, 37]. Although EMRs and decision support systems are used in different facilities, their use is rare in remote areas [27, 39].

The second type of solution, eHealth to facilitate care at a distance, relates to telemedicine like monitoring and communication systems between clinicians [38]. These systems offer services that provide care of similar quality to conventional care, but cost less [52]. Also known as telehealth systems, they allow the remote analysis of patient information by well-trained medical staff. Typical outcomes are improved diagnostic accuracy, reduced waiting times, and improved referral management [35, 36]. Remote monitoring systems have shown great potential for the management of chronic diseases [40]. Therefore, they can be applied in monitoring high risk pregnancies [26].

Outcomes in patient care from the use of health management systems, such as hospital management software, have not been measured. They have the potential to improve efficiency and reduce cost by improving logistics and the allocation of needed resources [53]. Furthermore, data about healthcare in remote areas can be gathered through these systems, increasing knowledge and facilitating research to improve MHC [38].

### Challenges to eHealth implementation

In this section, the different challenges to eHealth implementation are explained, based on the 5C model proposed by Dr. Peter Drury in [44]. Accordingly, they are divided into the five different components: context, content, connectivity, capacity, and community.

#### Context

Ehealth solutions must be adapted to the poverty context. Semi-structured interviews showed how health workers perform their jobs with very limited resources; they need to make the most of what they have. Therefore, the first step to providing solutions that have a positive impact on patient outcomes is understanding the needs of health workers. The ICT infrastructure assessment showed that remote areas face infrastructure problems such as poor or non-existent roads, limited access to electricity, and lack of telephone lines. In addition, high temperatures, humidity, and dust are prevalent in these communities. Regarding funding, the adoption of the SDGs by the Ghanaian Government leaves few financing opportunities for eHealth projects. All of these factors need to be considered before designing an eHealth solution.

#### Community

Rural communities in Kpando are characterized by low education levels and strong cultural beliefs. During the interviews, it was identified how these characteristics highly affect the opinion and attitude towards maternal health services. Pregnancy is not seen as a risk, and insufficient information is available on safe practices during pregnancy. Moreover, both field and literature showed how the decision to attend healthcare is influenced by family and previous experience. Thus community characteristics have to be considered in the design and implementation process.

Previous research demonstrated that the decision about what facility to attend is influenced by health workers and facility reputation [10]. The reputation is based on previous experiences of friends or family and direct knowledge of the staff. In the geographical area of this study, TBAs and traditional medicine are common. The community highly respects and trusts the practice of TBAs. They are easy to reach, and their services are affordable [8]. During the focus group discussions, TBAs explained how GHS had stopped providing them with training. In these discussions, other stakeholders (mothers and fathers) agreed that providing training to TBAs could be the key to improving MHC in islands and remote communities.

Previous research showed that women who receive MHC are generally satisfied with the care [46]. However, literature and field research showed how stigma toward teenage pregnancies and single women negatively influences the attitude of health workers toward their patients. This affects the information patients share with health workers. For instance, health workers provided examples of how patients might hide information about previous complications and sexually transmitted infections (STIs).

#### Capacity

The 5C model refers to capacity as technical capacity (i.e. infrastructure available), and manpower capacity, meaning not only the amount of human resources available but their skills (both technical and medical) [44]. Around 80% of the interviewed health workers had basic IT skills and own smartphones. However, training will be needed for the effective deployment of an eHealth solution. Unfortunately, staff rotation between facilities is common (as was observed during the research period), which could be a challenge for the effectiveness of this training.

The ICT infrastructure assessment showed that infrastructure is limited. Medical and IT equipment is often insufficient in CHPS zones. Technical support is not available, even in urban areas. Health workers pointed out broken equipment that could not be fixed due to the lack of technical support. When health workers were asked about community outreach, they all pointed to a lack of healthcare personnel. Therefore, outreach programs are conditioned to the number of patients that attend the facility at a given time. Two health workers even gave real examples of how the lack of doctors had recently led to maternal deaths.

#### Connectivity

Lack of wired networks is the main challenge for connecting rural and urban areas. In rural areas, network coverage and mobile phone penetration provide new connectivity opportunities. However, the coverage in rural areas is very unstable and varies between operators. Network coverage was measured in the selected health facilities and communities. The results show that 2G and 3G are available in 86% of the facilities; however, 3G is available 20% of the time, while 2G is available 76% of the time—and 4% of the time no connection is available. Facilities in urban areas have good mobile connections to allow video and web communications, but in rural areas the capacity is limited to web browsing. Nevertheless, web browsing typically requires more bandwidth than most eHealth applications. Thus, the bandwidth of rural areas should often be enough. Blind spots, where even SMS and phone calls are difficult, are an additional problem. Nevertheless, the ‘blind spot’ limitation occurs frequently in hospital workplaces worldwide.

#### Content

A key factor identified during the interviews is how different facilities follow different protocols. Sometimes the care provided is not evidence-based; it is common to base diagnosis only on the answers to standard questions, without physical examination. Furthermore, the official referral guideline systems are not always well applied.

Health workers identified lack of structure and unavailable data about pregnancy complications as negatively influencing the care provided. The relevant information is necessary for appropriate diagnostics. However, in isolated communities, low attendance at the facilities and poor record-keeping results in relevant information being seldom recorded.

### Solution outline

**Fig. 6:**
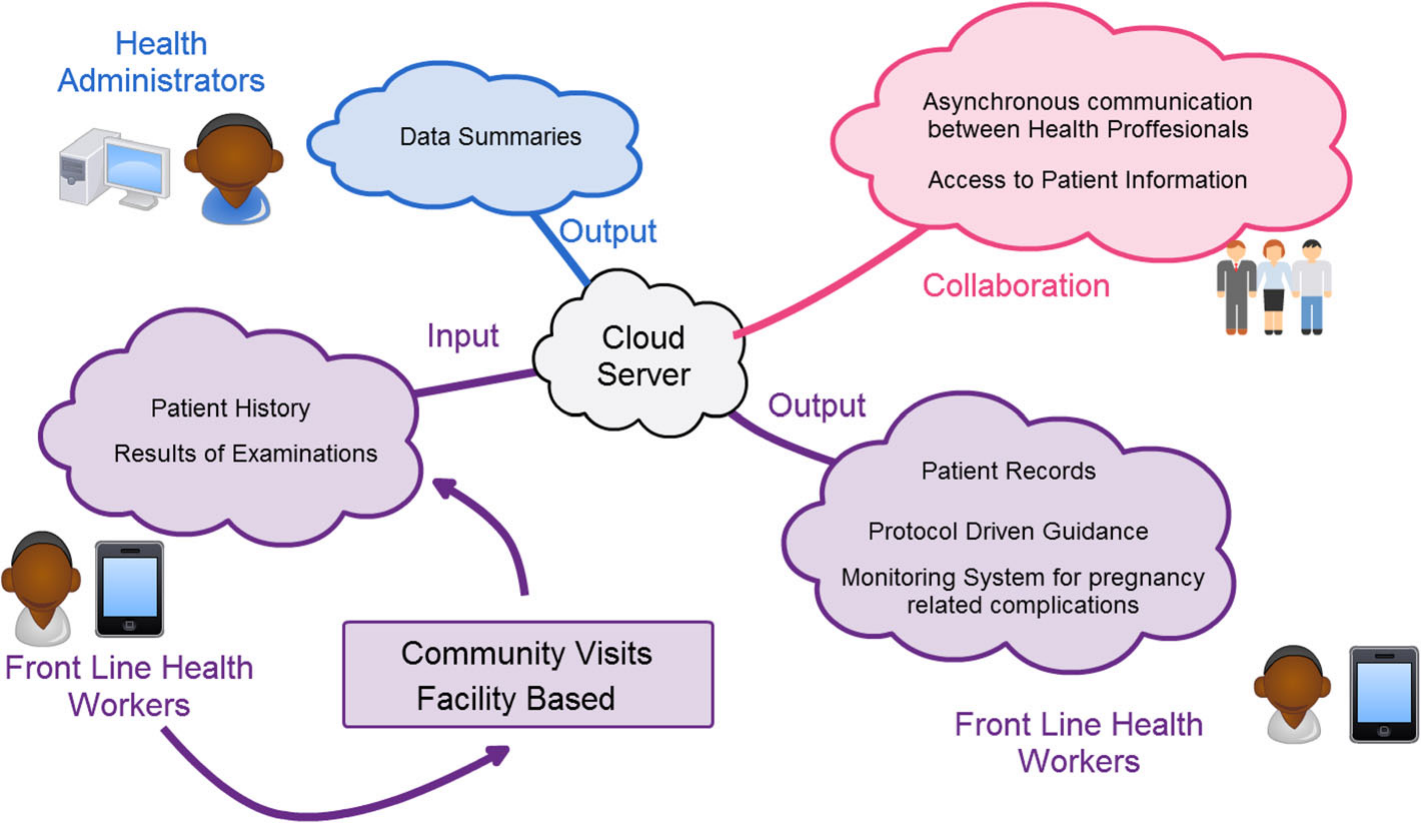
Proposed solution that addresses health workers and patients

This subsection outlines an eHealth solution that might optimize efficacy and feasibility based on the findings this work. Additionally, it might address the some of the main needs in MHC for patients and health workers in the situation considered in this work. Moreover, it is designed to fit under the current work practices of Kpando and areas with similar characteristics. A schematic is presented in Fig. 6. This solution, falling within the field of mHealth, is an application for Android mobile phones or tablets handled by front line health workers (CHNs in CHPS zones). It is cloud-based and uses the mobile communication networks available in the facilities.

The solution should be focused on providing a pregnancy monitoring system and bridging the distance between facilities and communities. It has the potential to make a substantial impact in the third delay and solve direct challenges in content and context as defined by the 5C model. To fulfill this potential, decision support systems and data gathering services need to be included in the application; they would help develop evidence-based birth plans to increase skilled maternal health attendance. The solution should be adapted to fit into the workflow of healthcare facilities in order to be used in facilities and outreach programs. Equipping CHNs with mobile phones or tablets and basic medical equipment to monitor pregnancies would be a requirement.

The application would allow facilities to share information, improving patient tracking and collaboration between professionals. It would provide health administrators with access to data summaries, which could improve reporting and logistics systems — addressing the content and capacity of the 5C model. The system should be designed to work offline, asynchronously sending the information to the cloud server whenever a connection is available. Cloud services are accessible in Ghana and allow remote maintenance of the network, a feasible solution to reduce connectivity challenges.

## Discussion

This paper assesses the feasibility, in terms of potential of and requirements, of eHealth solutions to improve maternal healthcare in remote areas of Kpando, Ghana. The MHC situation in Ghana faces different challenges than the ones seen in the developed world. There are challenges that affect the demand side, i.e. the patient, and others that affect the supply side, i.e. the healthcare provider. Factors on the demand side are personal, and often cannot be addressed by an eHealth solution. Otherwise, an eHealth solution can help solving challenges on the supply side. This way, although an eHealth solution cannot decrease stigma, or improve road infrastructure, a well-designed solution can be used to improve quality of care. Further, eHealth solutions can provide a new healthcare delivery model that will reduce delays in receiving adequate maternal healthcare. However, these solutions need to be adapted to the local context. Hence, the importance of identifying potential and requirements of eHealth implementation.

Our findings on MHC challenges on the supply and demand side are in line with the challenges in the medical system indentified in [15]. In [15] authors present the challenges of maintaining a residency traning program in rural Ghana. The slight differences in findings are that authors in [15] focused on identifying challenges in the whole medical system and not only in MHC. In addition, our study also focused on identifying those challenges on the demand side. Financial dependence of women, was identified as main challenge on the demand side in our study, while [15] also finds it as a key barrier for accesing medical care, especially since NHIS (free for pregnant women) does not cover the cost of all examinations and some women cannot cover the cost of transportation to the city to sign up.

Besides financial factors, we found cultural factors and social stigma key findings to explain low ANC attendance. ANC visits are important to early detection and prevention of pregnancy related complications. Thus, patient reluctance to seek treatment greatly increase the risk of complications. On the supply side, the most important challenge identified might be the lack of information about previous complications and patient history, which directly affects the standar of care provided and might lead to high risk pregnancies being treated in facilities that are not prepared to treat high risk cases.

Implementing eHealth solutions for rural communities in developing countries requires different approaches and strategies than in the developed world. There is much literature available with recommendations and guidance for eHealth implementation in developing countries. Their findings are consistent with the ones in this work [15, 19–21]. The main challenge for eHealth implementation is to achieve a high level of acceptance (in developing as well as in high income countries). Thus, community characteristics need to be considered in the design and implementation process of a solution. And important finding that might be a big cultural challenge, is the avoidance of STI discussion by patients. Further, connectivity and lack of infrastructure is a challenge in underserved rural areas of developing countries. Thus, solutions should be tailored to work under the local conditions and be robust to connection failures. For that reason, solutions that need synchronous communication between facilities or specialists are less effective than those that can work offline. Nevertheless, connectivity is expected to greatly increase in the future, which could solve these problems.

One of the challenges an eHealth solution field implementation could face is lack of trained personnel to use it. Thus there is a need for continuous and effective training, as well as planning ahead for the possibility of staff rotations. On the other hand, the fact that 80% of interviewed health workers own smartphones is a facilitator for mHealth solutions. Moreover, it is likely that eHealth solutions will face technical problems. Difficulties with the replacement and/or repair of broken equipment need as well to be accounted for. Addressing this issues cloud services can provide remote management. Another solution is to use technology from companies that have technical support across the country. These findings are in line with the ones of [20, 21].

Finally, results might indicate that an eHealth solution will have the highest impact by providing accurate clinical information. Clinical information is crucial for the prevention and early detection of pregnancy complications. By this logic, a potential mHealth solution has been proposed that takes into consideration all the findings of this study. If the proposed solution is able to surmount these obstacles, it could have a high impact in the area, as well as on other areas of similar characteristics.

### Strengths and limitations

Among the strengths of the research, are its qualitative design and the inclusion of many communities and stakeholders. The latter adds to the conceptual generalizability of the results to other remote areas in Ghana and neighboring countries. The qualitative setup of the study permitted the research objective to be examined in depth and comprehensively, allowing subtleties and complexities to be discovered.

On the other hand, one limitation is the cultural differences (including language barrier) between participants and researchers. These differences could have created response bias towards socially desirable answers; some participants may have been reserved towards the researcher in some aspects of their answers. Culture differences may also have led to misapprehension of sensitive and subtle topics during analysis. Further, interviews were not recorded since participants did not feel comfortable to be recorded due to personal and professional issues. Consequently, misinterpretation of questions and answers may have altered the true meaning of some discussed subjects.

Triangulation of different research methods and data, with the inclusion of different stakeholders, was used to increase the validity of this study. Moreover, the incorporated member checks and the active seeking of negative cases increased understanding of the stakeholders.

## Conclusions

Maternal healthcare in rural areas in Ghana is affected by three delays that influence the outcomes of care. These delays are influenced by socio-economic factors, accessibility of the facilities, and quality of care provided. One of the factors that lower quality of care is that health workers in remote areas are not trained to monitor high-risk pregnancies. Socio-economic factors include social stigma and traditional beliefs, which are strongly embedded in Ghana. These factors, along with deficient transportation means, influence maternal skilled care attendance.

Ehealth solutions show great potential to improve the factors that influence the delays. This is partially thanks to the high number of health workers with basic IT skills. However, identified challenges according to the 5C model need to be taken into account when designing an eHealth solution. Main challenges are the lack of infrastructure and technical support, the decision making process of patients based in cultural beliefs and previous experiences, poor mobile connectivity, environmental challenges and the poverty context in general. Based on the 5C model, an eHealth solution was outlined aiming to make the highest impact on improving guidance during the pregnancy.

The challenges and facts presented in this research can be used as background guidelines for other eHealth solutions to improve maternal health in Ghana. Furthermore, the conclusions of this article can be extrapolated—with caution—to other rural areas and remote communities in Africa.

### Future work

A mHealth solution has been outlined, based on health workers’ needs as identified during the research. In the next steps, the solution will be prototyped and re-designed following a human-centered approach. The aim is to create a solution that health workers will be willing to use.

In a following publication the mHealth solution will be described including aspects such as networking, security specifications, backup systems, and privacy protocols. These aspects will be assessed and incorporated into the design of the solution.

## Abbreviations

ANC: Antenatal Care
CHN: Community Health Nurse
CHPS: Community Health Planning and Services
CPR: Contraceptive Prevalent Rate
EmOC: Emergency Obstetric Care
EMR: Electronic Medical Records
GHS: Ghana Health Service
HC: Health Center
ICT: Information and Communication Technology
MDGs: Millennium Development Goals
MHC: Maternal Healthcare
MMR: Maternal Mortality Ratio
NHIS: National Health Insurance Scheme
PNC: Postnatal Care
RCH: Reproductive and Child Health
SBA: Skilled Birth Attendants
SDGs: Sustainable Development Goals
SMI: Safe Motherhood Initiative
TBA: Traditional Birth Attendants
TDM: Three Delays Model
UNiTED: Unifying Neighbors through Education and Development
WHO: World Health Organization
